# Visual Reliance for Balance Control in Older Adults Persists When Visual Information Is Disrupted by Artificial Feedback Delays

**DOI:** 10.1371/journal.pone.0091554

**Published:** 2014-03-10

**Authors:** Ting Ting Yeh, Tyler Cluff, Ramesh Balasubramaniam

**Affiliations:** 1 Sensorimotor Neuroscience Laboratory, Department of Kinesiology, McMaster University, Hamilton, Ontario, Canada; 2 Laboratory of Integrative Motor Behaviour (LIMB), Centre for Neuroscience Studies, Queen's University, Kingston, Ontario, Canada; 3 Cognitive and Information Sciences, University of California Merced, Merced, California, United States of America; University of Muenster, Germany

## Abstract

Sensory information from our eyes, skin and muscles helps guide and correct balance. Less appreciated, however, is that delays in the transmission of sensory information between our eyes, limbs and central nervous system can exceed several 10s of milliseconds. Investigating how these time-delayed sensory signals influence balance control is central to understanding the postural system. Here, we investigate how delayed visual feedback and cognitive performance influence postural control in healthy young and older adults. The task required that participants position their center of pressure (COP) in a fixed target as accurately as possible without visual feedback about their COP location (eyes-open balance), or with artificial time delays imposed on visual COP feedback. On selected trials, the participants also performed a silent arithmetic task (cognitive dual task). We separated COP time series into distinct frequency components using low and high-pass filtering routines. Visual feedback delays affected low frequency postural corrections in young and older adults, with larger increases in postural sway noted for the group of older adults. In comparison, cognitive performance reduced the variability of rapid center of pressure displacements in young adults, but did not alter postural sway in the group of older adults. Our results demonstrate that older adults prioritize vision to control posture. This visual reliance persists even when feedback about the task is delayed by several hundreds of milliseconds.

## Introduction

Although we can stand on a crowded bus with little difficulty, standing balance involves complex interactions between our body and the environment, sensory information from our eyes, skin and muscles, and control by distributed neural circuitry. Understanding the role of sensory feedback in balance control is central to unraveling the complexities of the human postural system [1].

While standing, vision, proprioception and vestibular inputs provide information about the body’s orientation in the environment [2]. The contribution of these sensory modalities to the internal representation of the body’s orientation and equilibrium depends on how the central nervous system assigns weight to each sensory modality [3]. A number of studies have shown that the sensory receptors that monitor body orientation are less sensitive in older adults (see [4]; [5] for a review). This reduced sensitivity has been linked to falling [6] and overreliance on visual feedback [7]–[9], which can disrupt postural control when visual inputs are altered or unreliable [10]–[12]. In addition to reductions in sensory reliability, delays in the transmission of feedback from the lower limb can exceed several tens of milliseconds [13]. These feedback delays may be problematic because the neural circuitry engaged in postural control must rely on information from the past to correct balance errors [14], [15]. Despite evidence that sensory delays increase during aging [16], [17], it is unclear how these additional feedback delays affect standing balance.

Human postural sway is composed of two components: a slow, non-oscillatory component (low frequency), and a fast, damped-oscillatory component (high frequency) [18]. When we stand as still as possible, the majority of variance is in the slow component of postural sway, and may be linked to imperfect estimation processes involved in feedback control [19], [20]. One way to investigate how feedback delays influence postural control is to impose time delays on visual information about the task [21], [22]. We have used this technique to show that delayed visual feedback increases the variance of both the low and high-frequency component of postural sway in young adults [23], and have used these findings to construct a model of the time-delayed postural system [24]. However, it is still unclear whether delayed visual feedback influences postural control in older adults. The notion that older adults emphasize vision to correct postural errors [8], [9] leads to the hypothesis that visual feedback delays compromise posture to a greater extent in older adults than young individuals.

Numerous studies have used dual-task paradigms to investigate how cognitive [25]–[28] and motor tasks [29]–[31] influence balance control in young and older adults. The emerging consensus is that age-related decline in cognitive-attention function make it difficult for older adults to shift or divide attention in dual-task conditions [32], [33]. The inability to shift attention from postural control to secondary tasks [34], [35] may alter sway variability when older adults perform the cognitive task under delayed visual feedback conditions. Following directly from our previous work [23], we also predicted a reduction in the variability of high-frequency centre of pressure (COP) displacements in young adults in the cognitive dual-task conditions.

In this experiment, we imposed time delays on COP feedback to investigate the role of vision in postural control in healthy young and older adults. The postural task required that participants position a cursor representing their COP in a fixed target as accurately as possible in an eyes-open condition (no visual feedback about the COP), and in conditions where visual COP feedback was delayed by as much as 900 ms. In some conditions, subjects also silently performed an arithmetic task to examine the interplay between cognitive performance and postural control. The key feature of the task is the explicit goal to minimize COP deviations to stay within the postural target. Because postural sway compromises task performance, this feature enabled us to directly test how visual feedback delays and cognitive performance affect balance control in young and older adults.

We observed increases in the variability of low and high frequency postural corrections in delayed visual feedback conditions. Older adults’ COP displacements were substantially more variable than the group of young adults when time delays were imposed on visual COP feedback. Our method of delaying visual feedback provides new insight into feedback mechanisms involved in postural control in healthy young and older adults.

## Methods

### Participants

Healthy young (n = 14, age  = 23.5±3.2) and older adults (n = 14, age  = 72.4±4.7) participated in the study. The older participants were recruited from a local physical activity program where they exercise at least two days per week. The participants did not report any balance deficits, visual impairments, orthopedic or neurological disorders. The McMaster University Research Ethics Board approved the experimental procedures and participants provided written informed consent prior to the experiment. The participants could withdraw from the study at any time without penalty.

### Apparatus

**Figure 1:**
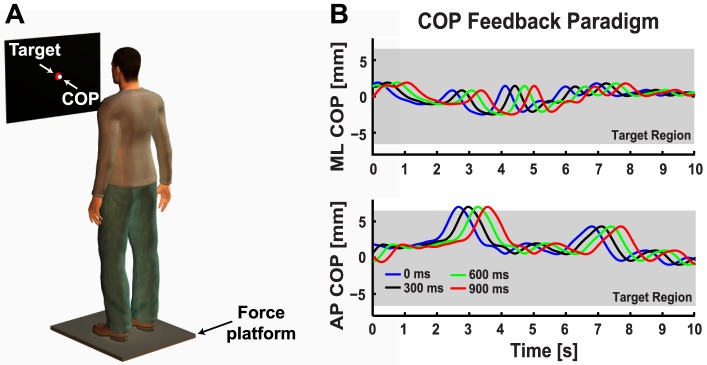
Experimental apparatus and delayed visual feedback paradigm. **A.** Schematic of the experimental setup showing the participant standing on the force platform in a comfortable shoulder-width posture with their arms placed at their sides. The visual display was located at eye level in front of the participant and displayed a fixed red circle (13 mm radius) corresponding to the target COP position. In the visual feedback conditions, a white cursor (10 mm radius) displayed the real time (instantaneous visual feedback, 0 ms delay) or delayed (300, 600, 900 ms visual feedback delay) COP position. Participants were instructed to position their COP on the fixed target as accurately as possible throughout the trial. **B.** Representative data taken from a single trial (31 seconds) performed by a young subject to illustrate how the feedback delay influenced the COP position shown on the visual display. Ten seconds of COP data are plotted to show the effect of the visual feedback delay. The blue traces are the true mediolateral (ML COP, top panel) and anteroposterior (AP COP, bottom panel) COP position displayed in the 0 ms visual feedback delay condition. The black (300 ms delay), green (600 ms delay) and red (900 ms delay) traces correspond to what was shown on the visual display in the delayed visual feedback conditions. Shaded grey region corresponds to the boundaries of the fixed COP target.

COP data were recorded from a force platform (OR6-2000, AMTI, Newton, MA, USA) positioned 1 meter in front of a 19 inch flat-panel LCD monitor located at eye level (Viewsonic, 60 Hz refresh rate, 5 ms delay; Figure 1A). COP data from the anteroposterior (AP) and mediolateral (ML) axes were sampled at 100 Hz (National Instruments DAQ PCI-6200) with MATLAB (7.9.0, The Mathworks, Natick, MA, USA) and stored offline for further analysis. Delayed visual feedback of the AP and ML COP position was displayed during the task using custom MATLAB code (see [24] for further details). The system gain was set such that a 1 cm COP displacement produced 1 cm of motion on the visual feedback display.

### Task and Procedure

At the start of each trial the visual display consisted of a fixed postural target (13 mm diameter red circle). We set the location of the target for each subject based on a 5-s window of quiet standing at the start of each trial. The target position was fixed in this location for the duration of the trial. We also used this initial COP data as a buffer to display the COP position at the start of the visual delay trials. We instructed participants to position their COP feedback cursor (10 mm diameter white circle) within the postural target for each 31 s trial.

The participants stood comfortably in a shoulder-wide stance with their arms placed at their sides. We marked each participant’s preferred foot placement on the platform to ensure it was consistent throughout the experiment. At the end of each trial, we removed the target and visual COP feedback, and the participant sat for a short break. We ensured the participant’s feet were lined up with their preferred foot placement before they began the next trial. Participants performed several practice trials before the experiment. During these practice trials, we instructed the participants to hold their COP cursor in the stationary target.

The experiment consisted of five conditions: eyes open (EO), and visual feedback about the COP position delayed by 0 ms (instantaneous feedback), 300, 600, and 900 ms. The visual display in the EO condition consisted of a stationary target with no visual feedback about the center of pressure. We instructed the subjects to focus on the center of the stationary target and stand as still as possible for the EO balance trials. The total display and machine processing delay were approximately 50 ms (determined using high-speed video analysis).

Figure 1B shows representative data from a single trial performed by a young subject in the 0 ms delay condition. We have illustrated how the delayed visual feedback paradigm influenced the COP-feedback display by plotting AP COP data shifted by 0 ms (blue trace, instantaneous COP feedback), 300 ms (black trace), 600 ms (green trace) and 900 ms (red trace). Increasing the visual delay caused a discrepancy between the true and displayed position of the COP. This enabled us to contrast the effect of visual feedback delays on posture control in young and older adults.

We used a silent arithmetic task to examine the interaction between postural control and cognitive performance [23], [25], [30], [31]. Prior to each trial, the participant was asked to remember a two-digit number. During the trial, the participant added or subtracted a single digit number from the running total at a rate of one calculation every 5s. An example series is: 53 (before trial)+2 (start of trial)-8 (5s)-2 (10s)+7 (15s)-1 (20s)-9 (25s)+3 (30s)  = 45 (30+s). The participants calculated the running total silently and verbalized their response after the trial was completed. Overall, the participants performed 3 trials in each condition (30 trials in total). We randomized the order of the ten conditions (5 visual conditions × 2 cognitive conditions) within each block of trials (10 trials/block). The total time to complete the experiment was ∼1hr.

### Data Analysis

We discarded the first 900 ms of COP data from each trial because it was considered the maximum length of the visual delay (+machine processing and display updating delays). The remaining 30.1s of COP data was used in the subsequent analysis. We calculated the time the COP was outside of the fixed target by comparing the radial COP position to the radius of the fixed target. The total time outside of the target was determined for each trial on a subject-by-subject basis and then averaged across trials for each condition. This measure allowed us to quantify performance in the postural task, and verify that subjects were engaged in the task and actively holding the postural target.

We performed the COP decomposition analysis by filtering ML and AP COP signals using a bidirectional, second-order Butterworth filter with an effective cutoff frequency of 0.3 Hz [22]. The 0.3 Hz cutoff frequency was selected based on work by van den Heuvel and colleagues [22]. The filter cutoff was also chosen based on the finding that visual stimulus motion induced a peak in the COP power spectrum at 0.3 Hz [36]. This study demonstrated that even in human postural control, which receives rich input from interacting sensory systems, a simple change in visual feedback alters the frequency composition of COP signals. We used high and low-pass Butterworth filtering routines to decompose COP data and compute the variability of low (< 0.3 Hz) and high frequency (> 0.3 Hz) COP displacements. The filter cutoff frequencies were adjusted to reduce signal power to 50% at 0.3 Hz [37]. Standard deviations were calculated from the filtered time series for each subject and then averaged to contrast COP variability between visual feedback and cognitive dual-task conditions. We also compared error rates in the cognitive task between the group of young and older adults.

### Statistical Analysis

Differences in time spent in the postural target were contrasted using a 2 AGE (Young vs. Old) × 2 COG (No Cognitive task vs. Cognitive task) × 5 feedback conditions (EO (no COP feedback), 0ms (no COP feedback delay), 300, 600, 900 ms) mixed-factor analysis of variance (ANOVA) with repeated measures on the cognitive task and visual feedback conditions. We performed the same statistical analysis on the standard deviation of low and high frequency ML and AP COP displacements. We used the Huyhn-Feldt correction factor when our data violated the sphericity assumption of the ANOVA test (Mauchley’s test, *p*<0.05). Significant interaction effects were analyzed using *post hoc* paired sample *t*-tests for each group separately, or independent samples *t*-tests between the two age groups. Bonferroni adjustments were applied to correct for multiple comparisons with the threshold significance level set at *p*<0.05 for each contrast. Throughout the manuscript, we report corrected *p*-values obtained by multiplying the *p*-value by the number of pairwise comparisons. In addition, we conducted an independent sample *t*-test on error rates to compare cognitive performance between the two age groups. Note that the small difference in baseline COP variability between the young and older adults does not jeopardize the conclusions of this study. Similar results were obtained when we normalized the data to COP variability in the EO condition with no cognitive task (please see Figure S1) and repeated the analysis.

## Results

### Performance in the postural task

**Figure 2:**
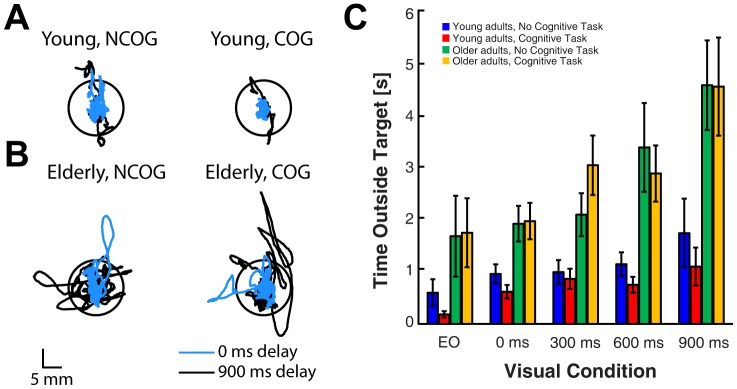
Comparison of COP displacements exhibited during simultaneous cognitive task performance and delayed visual feedback in young and older adults. A. COP data from a single trial performed by a representative young subject. Black circle corresponds to the COP target. Cyan traces show data from the 0 ms condition (instantaneous feedback) and black traces correspond to the 900 ms visual feedback delay condition. Representative COP data from the 0 ms and 900 ms delay conditions are shown with (right column) and without (left column) simultaneous cognitive performance. B. Same format as in A, but COP data are from a single trial performed by a representative older adult. C. Summary plot showing the time spent outside of the postural target. Error bars represent ±1 SEM.

Figure 2A-B shows unfiltered COP data from a representative young and older adult. For this example, we have plotted COP data from a single trial in the 0 (blue trace) and 900 ms delay (black trace) conditions when the posture task was performed with (right column) and without (left column) the cognitive task (i.e., arithmetic task). Our measure of task performance was the time spent outside of the postural target. This performance measure was influenced by a main effect of visual feedback delay (*F* (2.3, 69.4)  = 9.82, *p*<0.001; Huynh-Feldt correction) and a significant visual feedback delay × age interaction (*F* (4, 104)  = 2.9, *p*<0.05). *Post hoc* analysis revealed that increased feedback delays reduced the time that older adults spent in the postural target (*F* (2.52, 32.75)  = 6.97, *p*<0.01; 900<600, 300, 0 ms, and EO, all *p’*s<0.05). We found similar performance decrements for the group of young adults (*F* (4, 52)  = 3.37, *p*<0.05; 600 ms<EO, *p*<0.05). The interaction effect of COG × AGE (*F* (1, 26)  = 5.46, *p*<0.05) was also significant, and *post hoc* analysis revealed a significant increase in the time that young adults spent in the postural target during the cognitive task (*F* (1, 13)  = 6.88, *p*<0.05), but did not alter performance in the group of older adults (*F* (1, 13)  = 0.661, *p* = 0.47) (Figure 2C).

### Low Frequency COP Displacements

**Figure 3:**
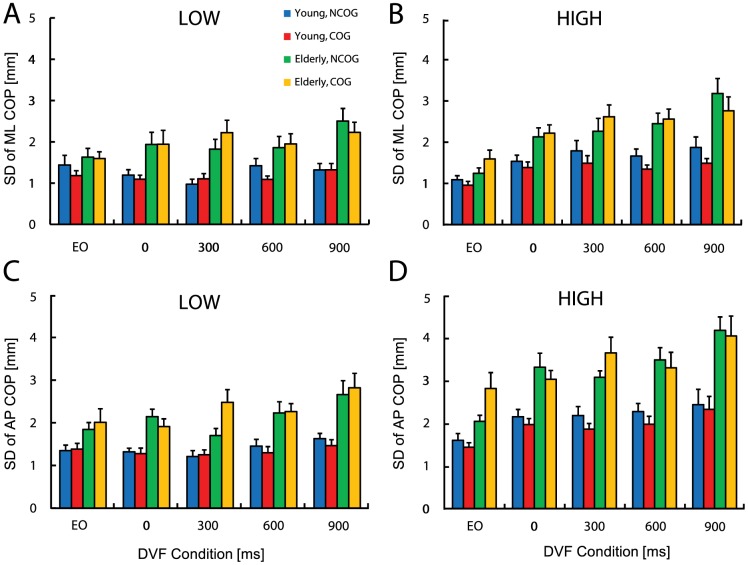
Summary plot of standard deviation of the LOW (left panel) and HIGH (right panel) ML (A, B) and AP (C, D) COP time series. Summary data are shown both with (COG) and without (NCOG) the cognitive dual-task for the eyes open (EO) and DVF conditions (0, 300, 600, 900 ms) in young and older participants. *Error bars* represent ±1 SEM.

We found that low-frequency ML COP displacements were influenced by the main effects of visual feedback delay (*F* (4, 104)  = 2.89, *p*<0.05) and AGE (*F* (1, 26)  = 11.54, *p*<0.01). Pairwise comparisons revealed that sway variability in the 300 ms condition was reduced relative to the 900 ms condition (*p*<0.01). In addition, sway variability for the young adults (*M* = 1.21, SE = 0.16 mm) was reduced compared to older adults (*M* = 2.00, SE = 0.16 mm). The interaction of visual feedback × AGE was also significant (*F* (4, 104)  = 2.96, *p*<0.05, Figure 3A), and *post hoc* analysis showed that older adults had greater sway variability across all visual delay conditions (0 ms: *t* (26)  = 2.53, 300 ms: *t* (26)  = 3.51, 600 ms: *t* (26)  = 2.43, and 900 ms: *t* (26)  = 3.75, all *p’*s<0.01), but not in the EO condition (*p* > 0.01). The cognitive task did not significantly alter the variability of low-frequency ML COP displacements (*F* (1, 26)  = 0.37, *p* = 0.55).

In the AP COP axis we found a significant main effect of visual feedback delay (*F* (2.32, 60.38)  = 5.42, *p*<0.01; Huynh-Feldt correction). Pairwise comparisons demonstrated that sway variability in the 0 ms and 300 ms conditions was reduced compared to the 900 ms visual delay condition (both *p*<0.01). Moreover, sway variability for the young adults was reduced compared to the older adults (*F* (1, 26)  = 23.90, *p*<0.001). However, the visual feedback × AGE interaction was not significant (*F* (2.32, 60.38)  = 1.64, *p* = 0.20; Huynh-Feldt correction, Figure 3C), and the cognitive task did not significantly alter the variability of low-frequency AP COP displacements (*F* (1, 26)  = 0.76, *p* = 0.39). Collectively, these results illustrate that visual feedback delays affected low-frequency postural displacements in both age groups, but importantly, that visual feedback delays increased sway variability to a greater extent in older adults.

### High Frequency COP Displacements

Figure 3B shows the variability of high-frequency ML COP data. The ANOVA revealed a significant main effect of visual feedback delay (*F* (3.13, 81.28)  = 17.20, *p*<0.001; Huynh-Feldt correction). Pairwise comparisons revealed that sway variability in the EO condition was reduced relative to the 0 ms, 300 ms, 600 ms, and 900 ms visual delay conditions (all *p*’s<0.01), and in the 0 ms relative to 900 ms condition (*p*<0.01). Sway variability for the young adults was reduced compared to the older adults (*F* (1, 26)  = 14.35, *p*<0.01). We observed a significant visual feedback × AGE interaction (*F* (3.13, 81.28)  = 2.84, *p*<0.05; Huynh-Feldt correction), with *post hoc* analysis revealing that compared to the young, older adults had greater high-frequency ML sway variability across all visual feedback conditions (EO: *t* (26)  = 2.12, and for the visual conditions: 0, 300, 600, 900 ms, all *t* values were greater than 2.12, all *p’*s<0.01). The visual feedback × COG interaction was also significant (*F* (2.94, 76.44)  = 3.06, *p*<0.05; Huynh-Feldt correction). For the young group, high-frequency sway variability in the EO (*t* (13)  = 2.66, *p*<0.01) and 600 ms conditions (*t* (13)  = 3.05, *p*<0.01) were reduced with the cognitive task. In comparison, the cognitive task did not significantly alter the variability of high-frequency ML COP displacements in older adults (all *p’*s > 0.01). We also found a trend that the young adults decreased high-frequency ML COP displacement under cognitive dual-task, whereas older adults showed the opposite. The interaction between cognitive task and AGE was marginally significant (*F* (1, 26)  = 4.19, *p* = 0.051).

**Figure 4:**
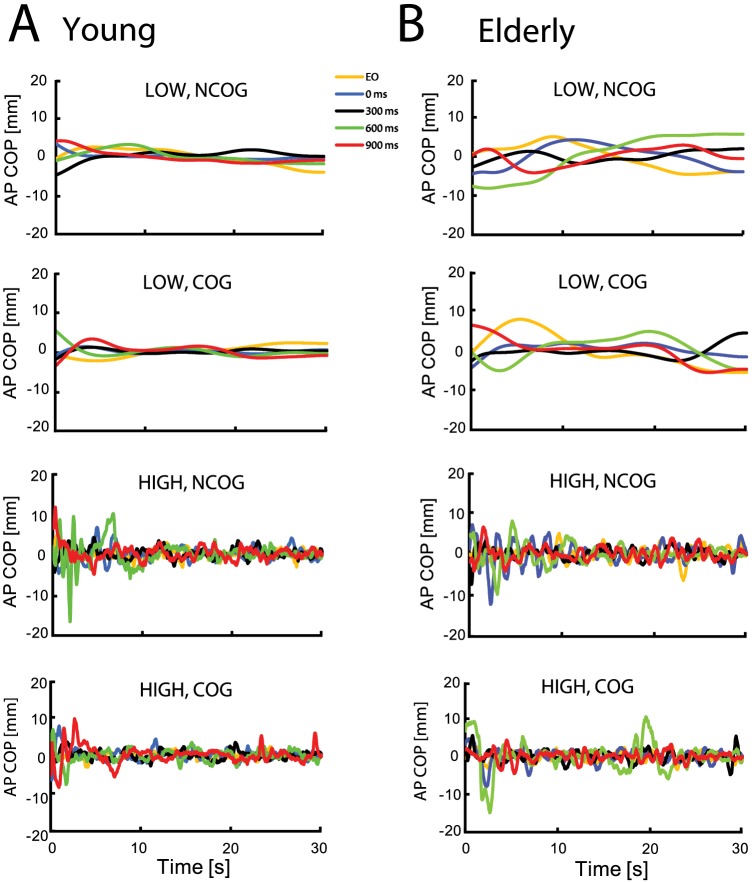
High and low-pass filtered anteroposterior (AP) COP displacements in delayed visual feedback and cognitive dual-task conditions. A. Representative low (LOW) and high-pass filtered (HIGH) AP COP time series from a single trial performed by a representative young participant in the visual feedback conditions with (COG) or without (NCOG) performing the simultaneous mental arithmetic task (Yellow: EO; Blue: 0 ms delay; Black: 300 ms delay; Green: 600 ms delay; Red: 900 ms delay). B. Same format as in A but from a single trial performed by a representative older participant.

**Figure 5:**
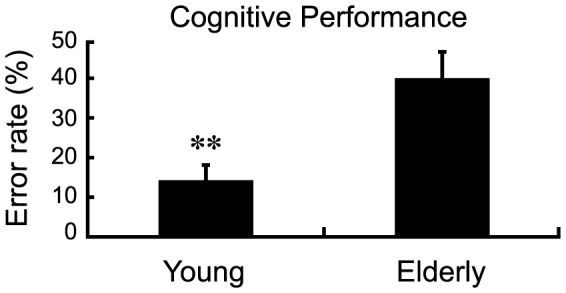
Cognitive performance in young and older adults. Cognitive performance was determined by error rate (numbers of errors/total number of questions*100%). *Error bars* represent ±1 SEM. ** *p*<0.01.

Figure 3D demonstrates that the variability of high-pass filtered AP COP trajectories was affected by the main effects of visual feedback delay (*F* (2.62, 67.99)  = 18.99, *p*<0.001; Huynh-Feldt correction) and AGE (*F* (1, 26)  = 19.85, *p*<0.001). Pairwise comparisons revealed that sway variability in the EO condition was reduced relative to the 0 ms, 300 ms, 600 ms, and 900 ms visual delay conditions (all *p*’s<0.01). Reduced sway variability was also noted in the 0 ms relative to 900 ms condition (*p*<0.01), as well as for the young adults compared to older adults. Finally, we found a significant three-way COG × AGE × visual feedback delay interaction (*F* (3.69, 95.93)  = 2.61, *p*<0.05; Huynh-Feldt correction). To examine this interaction, *post hoc* tests were applied separately for the young and older group. For the young subjects, the cognitive task decreased high-frequency AP COP in the 0 ms condition only (*t* (13)  = 2.42, *p*<0.01) (Figure 3D). In the older group, the cognitive task did not alter high-frequency AP COP variability in any of the visual conditions (all *p’s* > 0.05). A representative low and high-pass filtered COP data from the AP axis for a single trial performed by a young and older subject are shown in Figure 4. These results suggest that although young subjects exhibited a reduction in sway variability during dual-task postural control, postural control in older adults did not benefit from the cognitive task, and moreover, older adults made more errors in the cognitive task (*t* (26)  = 3.36, *p*<0.05, Figure 5).

## Discussion

Although it is well established that sensory feedback plays a critical role in postural control, less appreciated is that delays in the transmission and processing of sensory feedback can disrupt standing balance [14]. Here, we demonstrated that delayed visual COP feedback increased sway variability in a goal-directed posture task. The key finding was that compared to young subjects, older adults exhibited substantially larger COP displacements in delayed visual feedback conditions. Note that increased postural sway reflects compromised balance control because the goal of the task was to hold a COP-feedback cursor within a stationary target.

Another notable finding was that the cognitive task had different effects on COP variability between age groups. Specifically, the cognitive task improved the performance (decreased COP sway variability) of young participants, but did not alter older adults’ performance. Taken together, the present work supports and extends the notion that older adults may rely more on visual information to guide and correct posture – even when visual information about the task is delayed by several hundreds of milliseconds.

### Increased reliance on visual feedback for postural control in older adults

During our goal-directed posture task, we found that visual feedback delays caused an almost 25% (young adults: 22%, older adults: 28%) increase in sway variability compared to veridical feedback about the COP. These changes were linked to increased variability in both the slow and fast component of postural sway. Our interpretation of this finding is that delayed visual feedback may disrupt the relationship between the anticipated consequences of postural corrections and feedback displayed during the task. Indeed, a number of modeling studies have suggested that sway variance during unperturbed standing arises from estimation errors about the body’s orientation. In agreement with this idea, increases in visual information about standing balance reduce variance in the slow component of postural sway [19], [20].

There is also evidence that the central nervous system may rely on an internal model of the body’s dynamics to enable rapid postural corrections in the presence of errors and external perturbations [1], [38]. Our finding that altered visual feedback disrupts postural control and causes an increase in sway variability may be consistent with this idea. Delayed visual feedback may evoke uncertainty about the task by creating conflict between sensory feedback and internal predictions about the state of the body. In agreement with previous work highlighting that older adults rely more on unreliable visual feedback during postural control [9], our findings point out compromised postural control in the presence of time-delayed visual COP feedback. Similar results have been noted in studies where posture was destabilized by tendon vibration, but this instability decreased as young subjects learn to ignore proprioceptive feedback because it does not provide accurate information about the task [39]–[41]. Although the mechanism is still unclear, an interesting avenue for future research is whether adaptation to visual feedback delays can reduce postural instability in older adults.

Another striking result was that visual feedback delays decreased performance to a greater extent in older than younger subjects. One possible explanation for this finding is that age-related changes in peripheral sensory function cause older adults to rely more on visual information during postural control. Indeed, studies have suggested that normal aging prolongs sensory reweighting processes, and causes an increase in sway variability when visual feedback is altered during postural control [42]. In addition, older adults display persistent increases in postural sway when they are exposed to visual motion stimuli [11], [12], suggesting they are unable to suppress unreliable visual cues [11], [12]. Our results corroborate these findings and suggest that older adults inappropriately rely on time-delayed visual feedback about their COP, which in turn increases COP variability. It would be interesting to see whether our results extend to other sensory modalities such as time delays imposed on light-touch feedback during postural control [43], [44].

It should be noted that the visual reliance in older adults was more evident in the ML sway axis. Winter et al. [45] have suggested that when subjects stand in a shoulder-width stance (as used here), postural control in the AP axis involves muscular control of the ankle joint, and control in the ML axis is linked to muscular control of hip motion. In support of this idea, previous work has shown that young adults rely more on the ankle strategy during quiet standing, whereas older adults rely on the hip strategy [46], presumably to compensate for the loss of plantar sensitivity and tibialis anterior strength [47]. This directional difference in COP measures may be clinically relevant, as the amplitude of ML sway discriminates well between fallers and non-fallers, especially under dual-task conditions [48], [49].

In summary, older adults displayed substantial increases in sway variability when we imposed time delays on visual feedback about the location of their COP. Although these results are consistent with a growing number of studies that manipulated visual feedback and reported compromised postural control in older adults [50], [51], further work is necessary to outline the contribution of visual feedback in postural control. One method to investigate whether older adults rely more on delayed visual feedback is to measure postural responses to perturbations that randomly shift or disrupt visual COP feedback during the task. This method has been used to highlight the importance of visual feedback in upper-limb reaching movements [52]–[54], as well as to assess feedback corrections in postural control [42]. The use of visual perturbations may disambiguate visual overreliance in the elderly and provide valuable insight into age-related differences in balance control.

### Interaction between delayed visual feedback and cognitive performance stabilizes posture in young but not older adults

A key finding in this study was the reduction in fast postural deviations when young subjects performed the cognitive task. This finding replicates our work outlining the effect of visual feedback delays on postural control in young adults [23], and is also consistent with work highlighting reduced postural sway when young subjects engage in simultaneous motor and cognitive tasks [29], [55]. Our results also agree with the observation that sway variance is reduced when young subjects divert attention from postural control, which has been shown to reduce the amplitude and increase the frequency content of COP displacements [56]. One mechanism to reduce postural sway during dual-task conditions is to increase baseline muscle activity [57]. Muscle co-activation has been observed at the ankle joint during challenging dual-tasks, and may limit postural sway by increasing reflexive muscle activity [31], [58], [59]. This mechanism needs to be examined by measuring the activity of lower limb muscles during postural control with delayed visual feedback.

On the other hand, seemingly automatic motor tasks like standing may require additional cognitive resources in late adulthood due to reductions in sensorimotor [60] and cognitive-attention functions [32], [33]. This may cause an increase in postural sway when older adults engage in cognitive or motor tasks during standing balance. For example, Kang and Lipsitz [61] reported that cognitive and postural tasks increase the amplitude of postural sway, and Szczepańska-Gieracha and colleagues [62] reported a delay in the onset of rapid postural corrections when older adults performed a cognitive task during standing balance. In this study, we did not observe any significant changes in postural sway when healthy older adults performed the cognitive task during postural control, although older adults made more cognitive errors than the group of young subjects. This finding may suggest that older adults prioritize posture at the expense of cognitive performance in challenging dual-task conditions (i.e., ‘posture-first principle’, [27]). It is important to keep in mind that all the claims we make in this paper are restricted to the control of upright standing posture; extending this claim to all posture control would require comparison of performance in standing and seated postures.

There are some limitations with linear filtering that should be mentioned. We chose the Butterworth filter to decompose COP signals into low and high frequency components. In agreement with standard filtering methods [37], we corrected the cutoff frequency of the low and highpass filters so that the COP signal was at half-power at 0.3 Hz. We selected the cutoff frequency based on the observation that visual scene motion induces a peak in the COP power spectrum at 0.3 Hz [2], [36]. The Butterworth filter has the main disadvantage of a wide transition band, and given the low cutoff frequency, we found some attenuation of COP signals in the filter passband. However, we treated the data from young and older adults with the same filtering routines. Thus, the differences in sway noted for young and older adults must reflect the experimental manipulations. While this limitation certainly reflects a shortcoming of the Butterworth filter, it is unlikely to jeopardize the outcome of our study.

## Conclusions

This study revealed that visual feedback delays reduce balance performance in a goal-directed posture control task. The extent of postural variability caused by these feedback delays depended on age, with older adults exhibiting greater sway variability in delayed visual feedback conditions. Further investigation of the mechanisms underlying postural control in the presence of visual feedback delays, including perturbations, adaptation and sensory reweighting mechanisms, may help unravel the complexities of postural control in young and older adults. Rehabilitation strategies for older adults with balance problems should take into account the phenomenon of over reliance on visual feedback.

## Supporting Information

Figure S1 Summary plot of normalized variability of LOW (left panel) and HIGH (right panel) AP COP time series. Summary data are shown both with (COG) and without (NCOG) the cognitive dual-task for the eyes open (EO) and DVF conditions (0, 300, 600, 900 ms) in young and older participants. *Error bars* represent ±1 SEM.
(EPS)
